# Supplementary material for: Direct RNA sequencing reveals m6A modifications on adenovirus RNA are necessary for efficient splicing
Source: Nat Commun. 2020 Nov 26;11:6016. doi: 10.1038/s41467-020-19787-6 (PMC7691994; doi:10.1038/s41467-020-19787-6)
Supplement: Supplementary file 1 — Supplementary Information [file 41467_2020_19787_MOESM1_ESM.pdf]

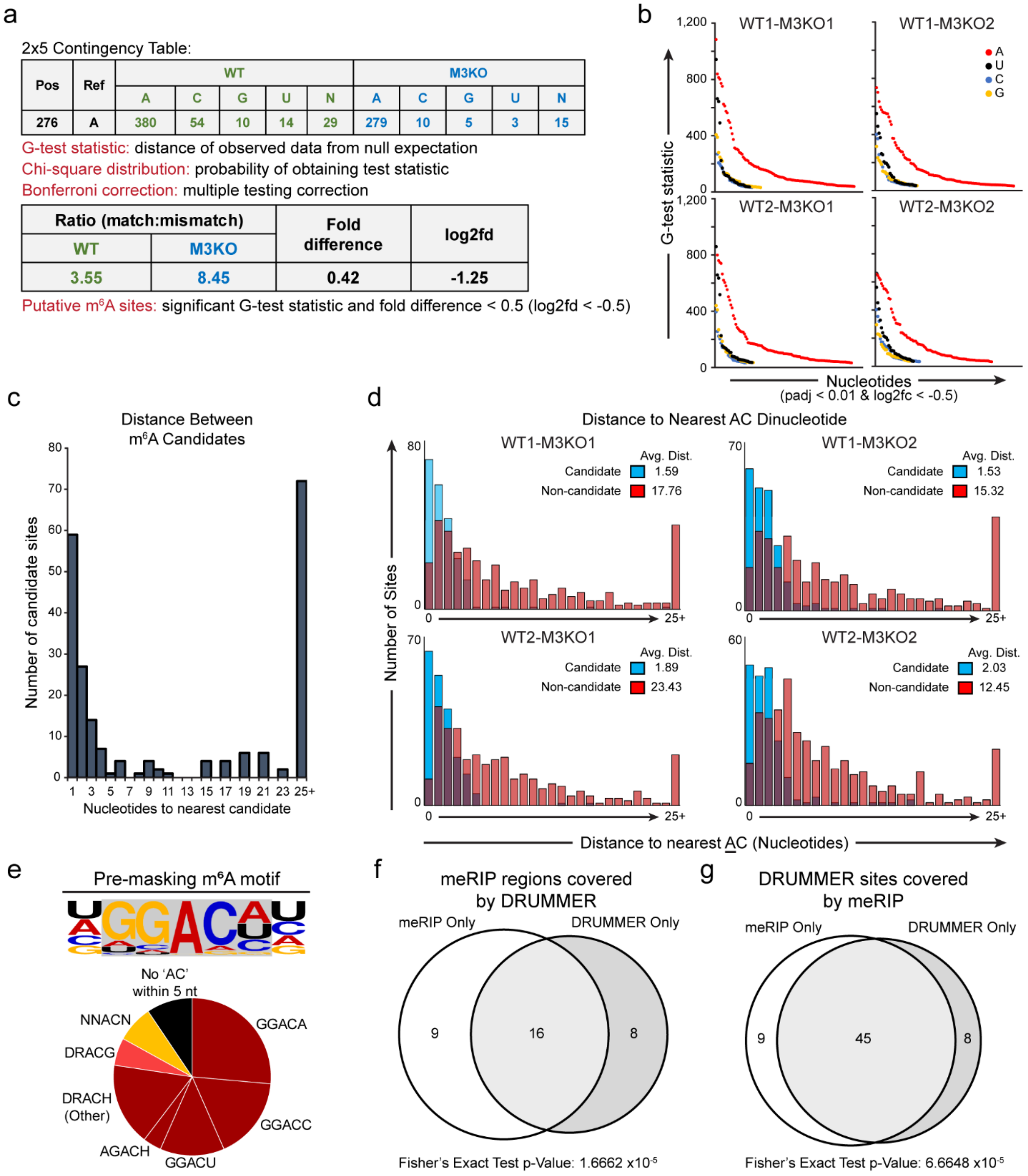

Figure S1. Direct RNA m^{6}A prediction clusters on AC dinucleotides. (a) Example 2x5 contingency table and statistical tests used to predict m^{6}A sites at nucleotide resolution by comparing the error rate between RNA generated in WT (METTL3-positive) versus METTL3 Knockout (M3KO) cells. **(b)** Distributions of the significant G-test statistic for each nucleotide with additional filtering for a one-fold or greater difference in the match:mismatch ratio between the m^{6}A positive (WT) and m^{6}A negative (M3KO) datasets. All G-test statistic p-values are corrected

for multiple comparisons using Bonferroni correction. **(c)** The distance (in nucleotides) between nearest neighbor candidates indicates that multiple candidate sites (significant G-test statistics) lie proximal to many putative m^6A modified bases. **(d)** Distance to the nearest A nucleotide in an AC dinucleotide within the Ad5 genome for candidate m^6A sites (blue) versus 200 randomly selected non-candidate sites (red). Average distance for the four distinct comparisons are shown in the legend. **(e)** The seven nucleotides surrounding the collapsed G-test m^6A candidates (before subsequent shifting to nearest 'AC' dinucleotides) reveal the canonical DRACN motif. **(f)** 16/25 meRIP peaks on the Ad5 transcriptome are recapitulated in the dRNA-derived m^6A candidates, a ratio that is higher than expected by chance based on two-tailed Fisher's Exact test. **(g)** 45/53 dRNA-derived m^6A candidates are covered by meRIP peaks on the Ad5 transcriptome, a ratio that is higher than expected by chance based on two-tailed Fisher's Exact test.

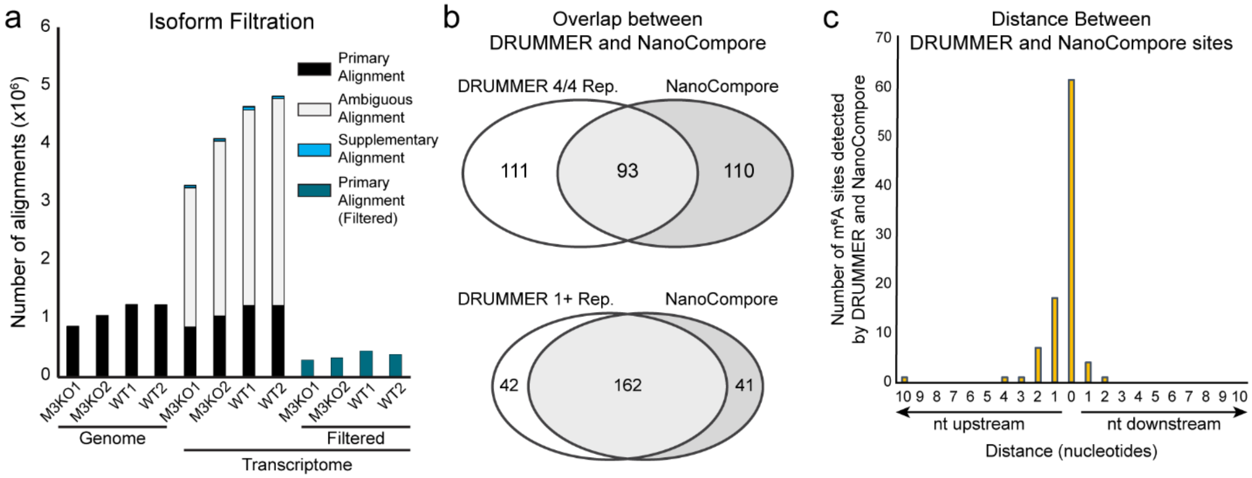

Figure S2. Comparison of DRUMMER and NanoCompore reveals significant overlap in m^{6}A site prediction. (a) Alignment of sequence reads to the adenovirus genome results in a single primary alignment per read (SAM flag 0). By comparison, aligning against the transcriptome can yield multiple primary alignments (termed secondary alignments, SAM flag 256, grey) and/or supplementary alignments (SAM flag 2048, blue). Subsequent filtering to retain only reads that unambiguously align to a single transcript (SAM flag 0, mapping quality >0, teal) results in approximately 70% of the sequence datasets being discarded. **(b)** NanoCompore yields 93/204 (45%) single-nucleotide m^{6}A candidate sites as our dRNA-based detection method, DRUMMER, when comparing sites detected in 4/4 of our pairwise comparisons (top). When this threshold is lowered to m^{6}A candidate sites detected in at least one DRUMMER comparison the overlapping ratio rises to 162/204 (79%, bottom). **(c)** DRUMMER and Nanocompore often identify the exact same nucleotide as potentially m^{6}A modified, with outliers detected within five nucleotides of one another.

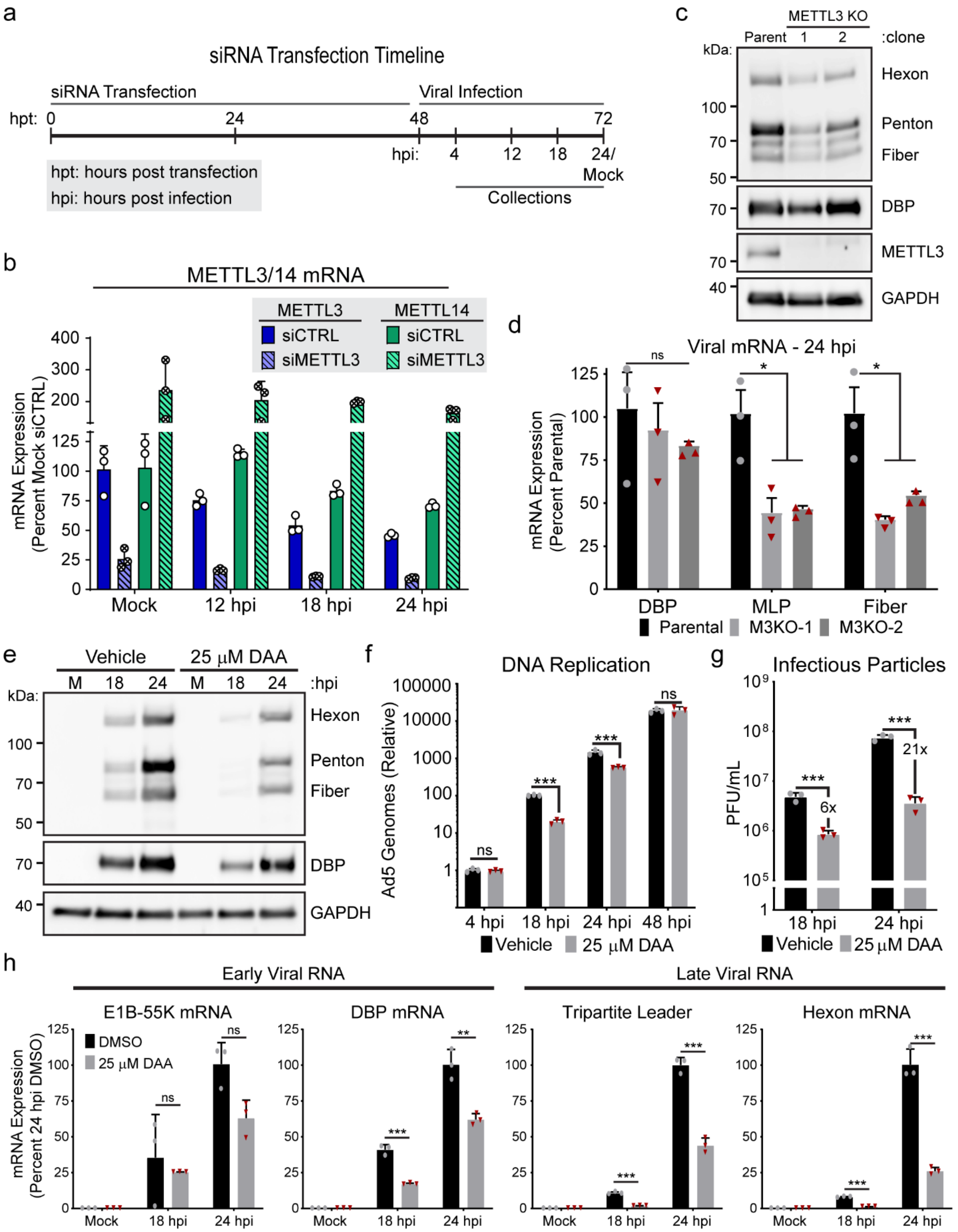

Figure S3. METTL3 knockdown is phenocopied by methylation inhibition or METTL3 knockout. (a) Schematic timeline of siRNA transfection experiments with the times of infection

and sample collection marked. **(b)** qRT-PCR showing levels of METTL3 mRNA (blue) or METTL14 mRNA (green) 48 hours post knockdown of control siRNA (siCTRL, filled bars) or METTL3 siRNA (siMETTL3, hashed bars). Each transcript is normalized to the abundance of siCTRL RNA at 48 hours post transfection (Mock) set to 100% and then monitored over the course of viral infection. **(c)** Two independent clones of Cas9-generated METTL3 knockout cells (M3KO) or the parental A549 cells were infected with Ad5 for 24 hours and analyzed for early protein expression (DBP) or late proteins (Hexon, Penton, and Fiber) by immunoblot. Immunoblot representative of three independent experiments. **(d)** Viral early and late RNA expression was measured by qRT-PCR at 24 hpi with Ad5. Samples include three cell culture triplicates of A549 Parental or M3KO clones 1 and 2. **(e)** Viral early and late protein expression was analyzed by immunoblot over a time-course of infection in cells treated with 25 μM m^6A-inhibitor 3-Deazaadenosine (DAA) or vehicle control. Immunoblot representative of three independent experiments. **(f)** Viral DNA replication was measured with qPCR in biological triplicate samples at different times after infecting A549 cells and concurrently treating with vehicle or 25 μM DAA. Fold-change in genome copy was normalized to the amount of input DNA at 4 hpi in vehicle treated cells. **(g)** Infectious particle production was measured by plaque assay at different times after infecting A549 cells and concurrently treating with vehicle or 25 μM DAA. **(h)** Viral RNA expression was measured by qRT-PCR in a time-course of infection after concurrently treating with vehicle or 25 μM DAA. Viral early transcripts are shown on the left, while late stage transcripts are shown on the right. Data include three biological replicates and significance was determined by unpaired two-tailed Student's T-test, * $p \le 0.05$, ** $p \le 0.01$, *** $p \le 0.001$, ns=not significant. Experiments are representative of three biological replicates and graphs show mean +/- standard deviation. Exact p-Values are included in the source data file.

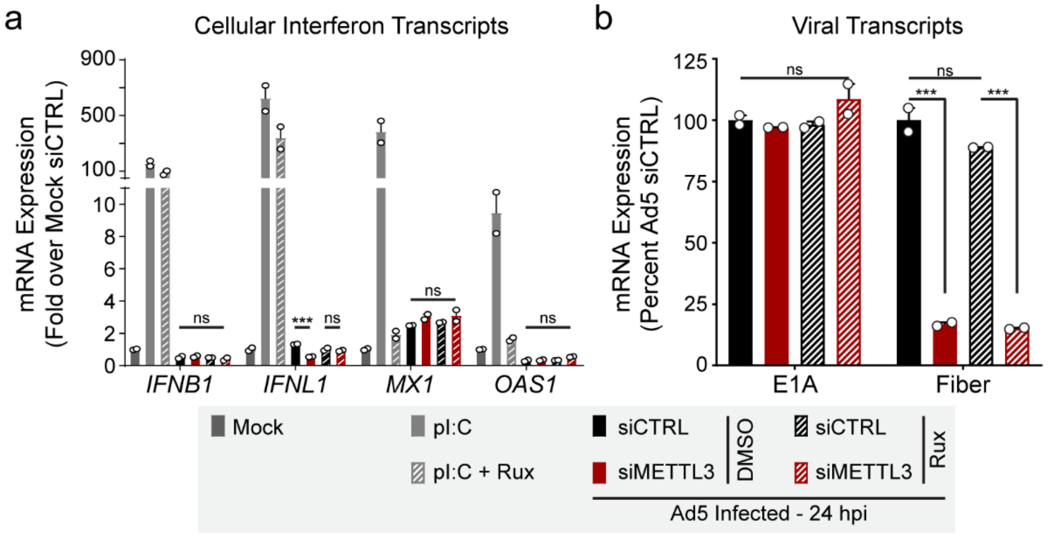

Figure S4. Interferon gene expression is not induced by METTL3 knockdown or adenovirus infection. (a) A549 cells were mock treated (dark grey bars) or transfected with the viral RNA agonist poly(I:C) for 24 hours (light grey bars). Concurrently, A549 cells were depleted of METTL3 with siRNA (red bars) or control treated with siRNA (black bars) before being infected with Ad5 for 24 hours. All sample were either treated with 4 μM Ruxolitinub (hashed bars) or vehicle control (solid bars) for the duration of infection or poly(I:C) transduction. Interferon RNAs were measured by qRT-PCR and displayed as fold-change over mock treated A549 cells. **(b)** Viral transcripts were quantified by qRT-PCR to assay expression of a viral early gene (E1A) or a viral late gene (Fiber). Data are representative of two biological replicates. For all assays, significance was determined by unpaired two-tailed Student’s T-test, *** p≤0.001, ns=not significant. Graphs represent mean +/- standard deviation. Exact p-Values are included in the source data file.

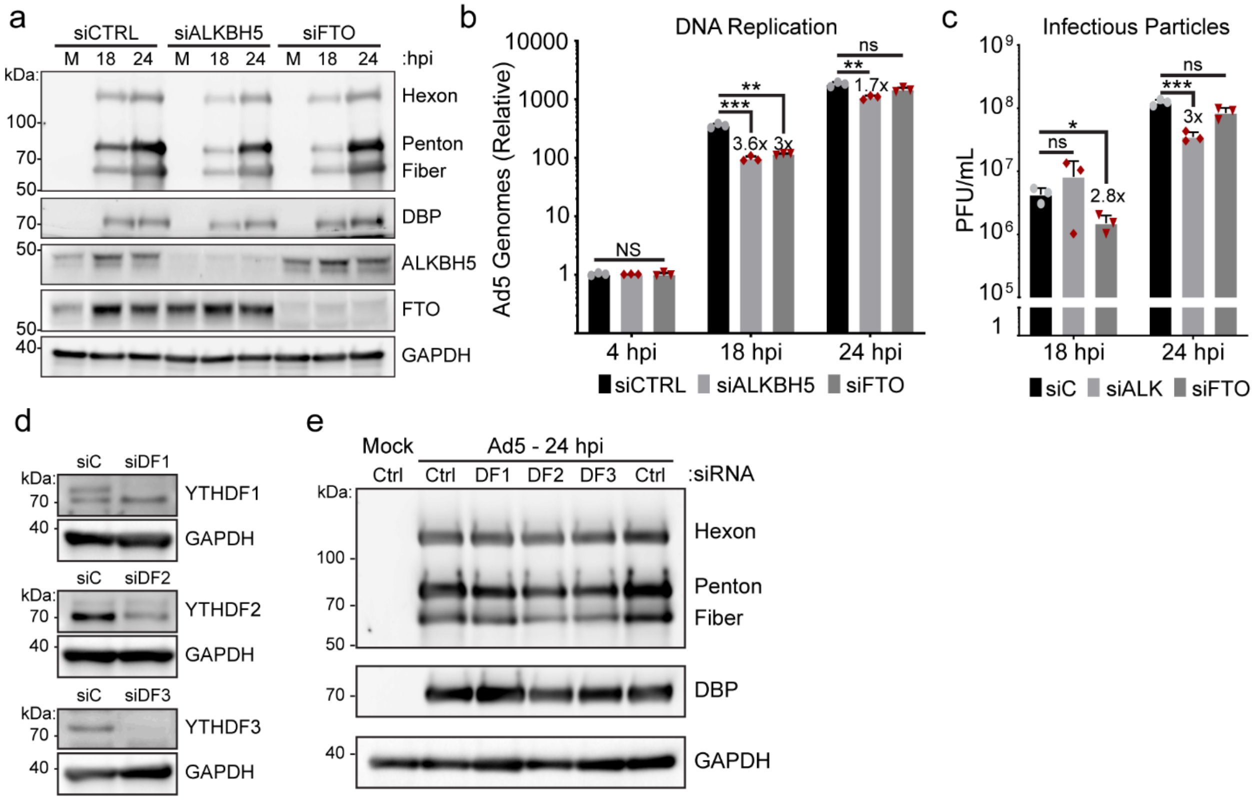

Figure S5. Cytoplasmic readers and m⁶A-erasers have minimal effects on the adenoviral infectious cycle. (a) A549 cells were transfected with control siRNA (CTRL), or siRNA targeting ALKBH5 or FTO for 48 hours before Ad5 infection. Viral late proteins were analyzed over a time-course of infection with polyclonal antibody against structural genes (Hexon, Penton, and Fiber) or the early protein DBP. **(b)** Viral DNA replication was measured in biological triplicate samples with qPCR over a time-course of infection in A549 cells depleted of ALKBH5 or FTO by siRNA. Fold-change in genome copy was normalized to the amount of input DNA at 4 hpi in control siRNA treated cells. **(c)** Infectious particle production was measured by plaque assay at different times post-infection after infecting A549 cells depleted of ALKBH5 or FTO by siRNA. Data includes three biological replicates. **(d)** siRNA-mediated knockdown of YTHDF1, YTHDF2, or YTHDF3 in A549 cells. **(e)** A549 cells were transfected with control siRNA (Ctrl), or siRNA targeting YTHDF1, -2, or -3 (DF1, DF2, DF3) for 48 hours prior to Ad5 infection. Viral early and late proteins were analyzed by immunoblot with the indicated antibodies. Immunoblots in panels **a, d,** and **e** are representative of three independent experiments. For all assays, significance was determined by unpaired two-tailed Student's T-test, * p≤0.05, ** p≤0.01, *** p≤0.001, ns=not significant. Graphs represent mean +/- standard deviation. Exact p-Values are included in the source data file.

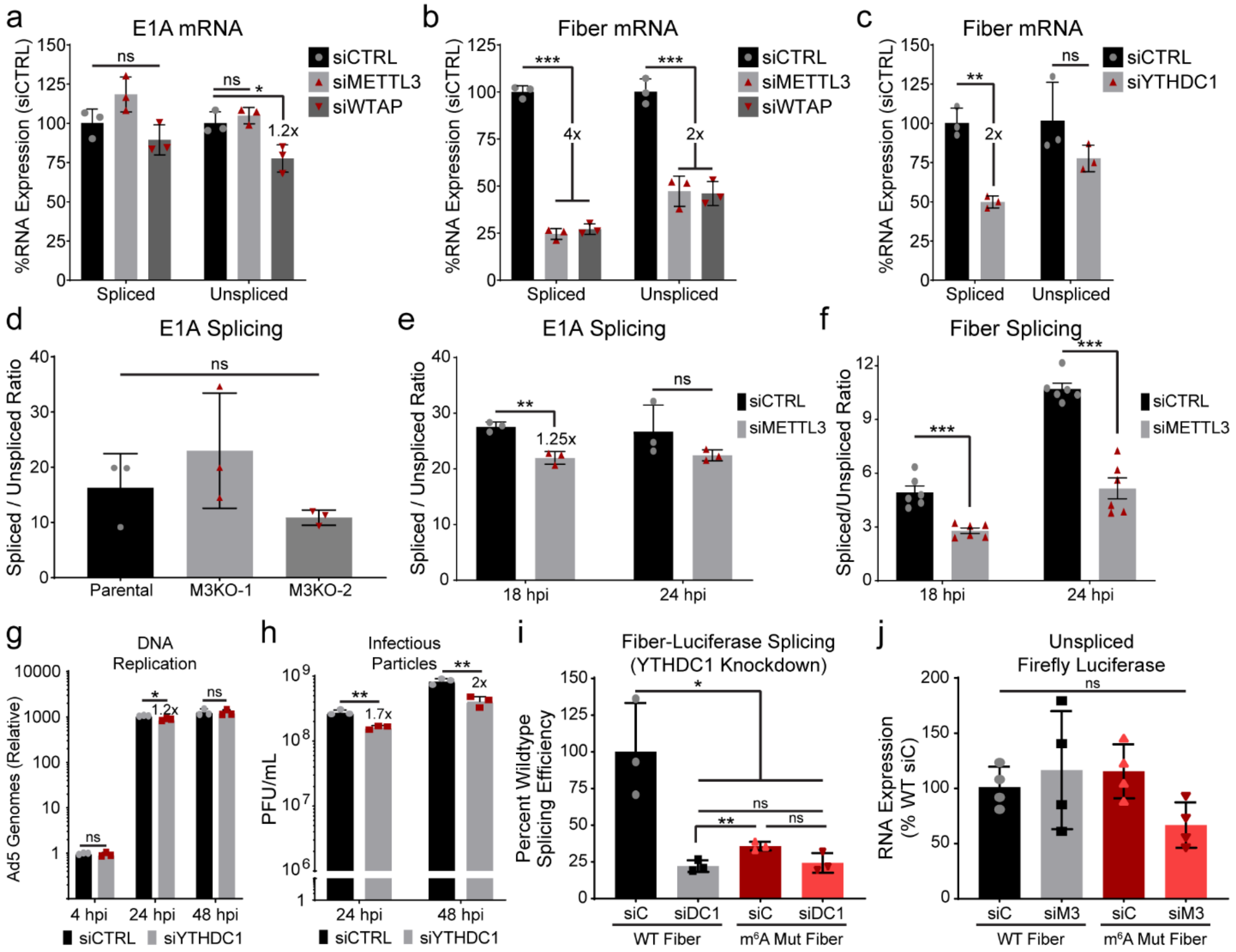

Figure S6. Splicing efficiency is mediated by YTHDC1 independent of time post infection. **(a)** Data from Figure 6d (E1A) split into the spliced and unspliced form of the transcript. **(b)** Data from Figure 6d (Fiber) split into the spliced and unspliced form of the transcript. **(c)** Data from Figure 6f split into the spliced and unspliced form of the Fiber transcript. **(d)** E1A splice efficiency was analyzed in Parental A549 cells or two independently derived METTL3 KO cell lines and represent three independent experiments. **(e)** The same splicing assay from Figure 6c was performed for E1A splicing efficiency at both 18 hpi and 24 hpi with Ad5 after knockdown of METTL3. Splicing efficiency data from 24 hpi are additional biological replicates of the data shown in Figure 6d. **(f)** Splicing assay from Figure 6c was performed for Fiber splicing efficiency at both 18 hpi and 24 hpi with Ad5 after knockdown of METTL3. Splicing efficiency data from 24 hpi are an additional six biological replicates of the data shown in Figure 6d. **(g)** Viral DNA replication was measured in biological triplicate samples with qPCR at different times after infecting A549 cells depleted of YTHDC1 by siRNA. Fold-change in genome copy was normalized to the amount of input DNA at 4 hpi in control siRNA treated cells. **(h)** Infectious particle production was measured at different times by plaque assay in biological triplicate after infecting A549 cells depleted of YTHDC1 by siRNA. **(i)** HeLa cells were control transfected (siC) or depleted of YTHDC1 (siDC1) by siRNA for 48 hours before transfection with either WT Fiber or m^{6}A Mut Fiber plasmid from Figure 6h. At 24 hours after the second transfection, splicing efficiency of the

transgene was assayed using Fiber-specific primers. Data represent three biological replicates. **(j)** PsiCheck2 plasmid used in Figure 6i and Figure S6j contains a control unspliced Firefly luciferase RNA expressed from a separate promoter. Total RNA was measured by qRT-PCR and normalized to the amount of transgene expressed from the WT plasmid in siCTRL treated cells. Data is representative of four biological replicates, and the lack of significance of variance was determined by ANOVA. For all other statistical assays significance was determined by unpaired two-tailed Student's T-test, * $p \leq 0.05$, ** $p \leq 0.01$, ns=not significant. Graphs represent mean +/- standard deviation. Exact p-Values are included in the source data file.

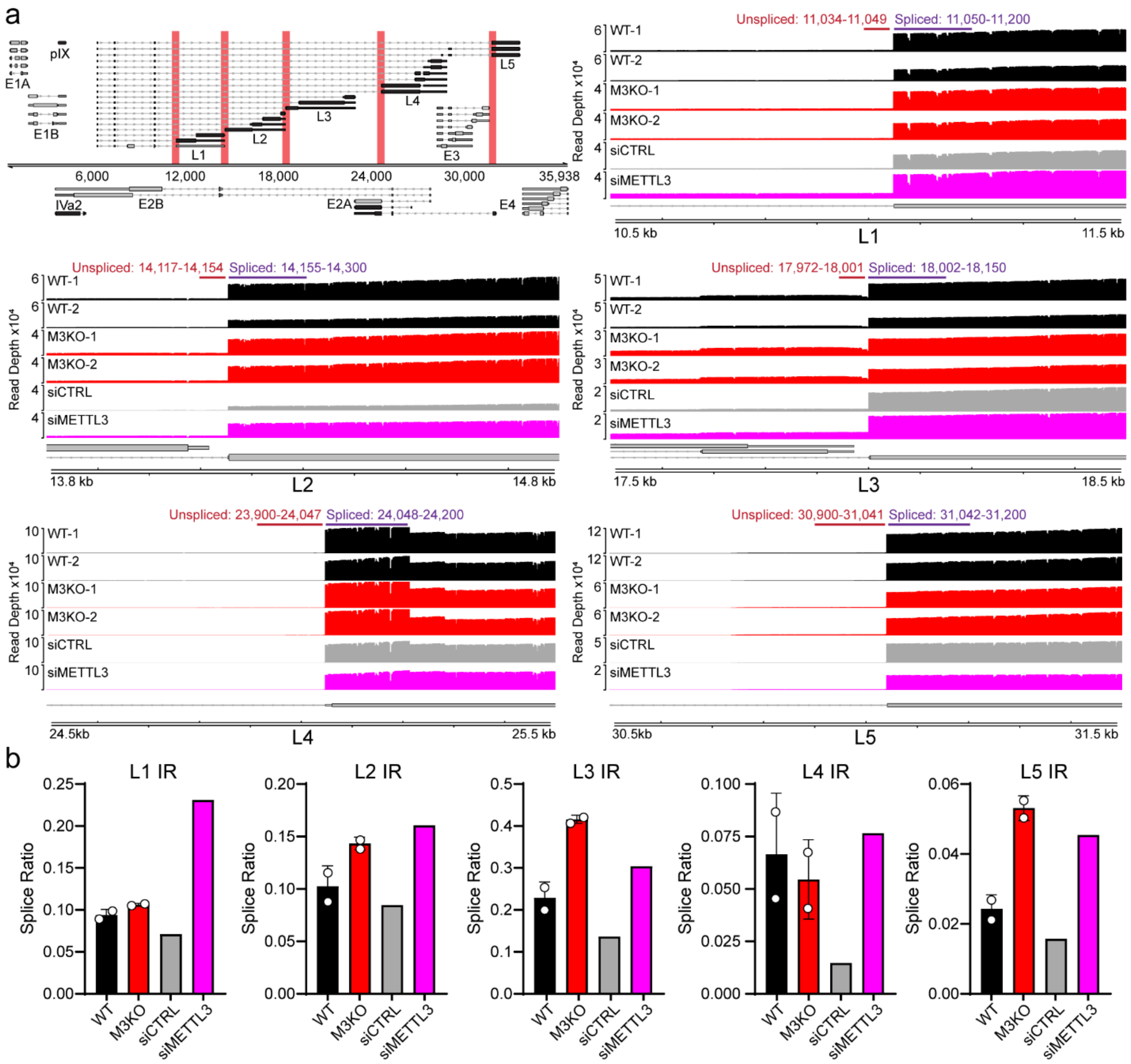

Figure S7. Long-read sequencing reveals increased intron retention of viral late transcripts upon METTL3 depletion. (a) (Top left) Regions of the Ad5 transcriptome analyzed for evidence of intron retention. Due to nanopore long reads starting at a specific 3' poly(A) site, transcripts within the individual L1-L5 regions defined by common polyadenylation were able to be binned and analyzed together. Representative genome viewer profiles of read depth within the targeted regions. Wildtype cells infected with Ad5 are shown in black (WT, two biological replicates), METTL3 KO infected with Ad5 shown in red (M3KO, two biological replicates), Ad5 infected control siRNA knockdown cells in grey (siCTRL, single replicate), and Ad5-infected METTL3 knockdown cells in magenta (siMETTL3, single replicate). For intron retention analysis, average read depth from spliced and unspliced windows (shown above each representative plot) were used. Size of windows were chosen to not overlap nearby genes. **(b)** Intron retention (IR) is measured for each late transcript region as a measure of splice ratio (average normalized unspliced read depth/normalized spliced read depth). Data represent the mean +/- standard

deviation of two biological replicates for Parent/M3KO and the single replicate from siRNA-mediated knockdown.

Supplementary Table 1. List of Oligonucleotides used in this study

	Name	Forward Primer (5' to 3')	Reverse Primer (5' to 3')
qRT Primers	Major Late Promoter Spliced	TTCCGCATCGCTGTCTG	CCGATCCAAGAGTACTGGAAAG
	Major Late Promoter Unspliced	GTCCAGGGTTTCCTTGATGAT	
	Fiber Spliced	GAAAGGCGTCTAACCAGTCA	AAAGGCACAGTTGGAGGAC
	Fiber Unspliced	CATCCGCACCCACTATCTTC	
	E1A Spliced	TGGACCCTCGGGAATGAA	TCAGGCTCAGGTTCAGACA
	E1A Unspliced	GGCTTAAGGGTGGGAAAGAA	
	Hexon	GAAAGGCGTCTAACCAGTCA	CCCGAGATGTGCATGTAAGA
	L4-100K		GGTTAGGCTGTCCTTCTTCTC
	E1B-55K	GAGCAGAGCCCATGGAAC	TGCCCATCCTCTGTAATTGTC
	E2B	CCATGGTCAAATGCTACCTGG	TCCTCGTCAGCGTAGTCTG
	DBP	ATCACCACCGTCAGTGAA	GTGTTATTGCTGGGCGA
	E4	GACAGGAAACCGTGTGGAATA	CACAGAGTACACAGTCCTTTCTC
	GAPDH	TGCACCACCAACTGCTTAGC	GGCATGGACTGTGGTCATGAG
	HPRT	TGACACTGGCAAAACAATGCA	GGTCCTTTTCACCAGCAAGCT
	MALAT1	CCGAGCTGTGCGGTAGGCATT	CGGTTTCCTCAAGCTCCGCCT
	IFNB1	CAGCATCTGCTGGTTGAAGA	CATTACCTGAAGGCCAAGGA
	IFNL1	CGCCTTGGAAGAGTCACTCA	GAAGCCTCAGGTCCCAATTC
	MX1	GGCCAGCAAGCGCATCT	TGGAGCATGAAGAACTGGATGA
	OAS1	GAAGGCAGCTCACGAAACC	AGGCCTCAGCCTCTTGTG
	Luciferase	GAGGTTCCATCTGCCAGG	CCGGTATCCAGATCCACAAC
Genomic Primers	DBP	ATCACCACCGTCAGTGAA	GTGTTATTGCTGGGCGA
	Tubulin	CCAGATGCCAAGTGACAAGAC	GAGTGAGTGACAAGAGAAGCC
	METTL3	CCGCGTGAGAATTGGCTAT	AAAGGTTCATTCCTCGCTTACT
Cloning	METTL3 into BB726	taccgcgggcccgggatccaATGGATTATAAGGATGATGATG	ctgcacctgagtgtttacttCTATAAATTCTTAGGTTTAGAGATG
	METTL3 PAM Mutant	GGACTCTCTGCGCGAGAGGCTGCAG	CTGCAGCCTCTCGCGCAGAGAGTCC
	METTL3 sgRNA px330	caccgGAAGCAGCTGGACTCTCTGC	aaacGCAGAGAGTCCAGCTGCTTCc
	psiCheck2 3'UTR	TCGAGTTACCGGTTCGC	GGCCCGGAACCGGTAAC
	Fiber Intron	TGAGGAGGCTTTTTTGGAGG	ACCTTGGAAGCCATGGTGG
