# Supplementary material for: Direct RNA sequencing reveals m6A modifications on adenovirus RNA are necessary for efficient splicing
Source: Nat Commun. 2020 Nov 26;11:6016. doi: 10.1038/s41467-020-19787-6 (PMC7691994; doi:10.1038/s41467-020-19787-6)
Supplement: Supplementary file 3 — Reporting Summary [file 41467_2020_19787_MOESM3_ESM.pdf]

nature research

Corresponding author(s): Matthew Weitzman

Last updated by author(s): Oct 5, 2020

Reporting Summary

Nature Research wishes to improve the reproducibility of the work that we publish. This form provides structure for consistency and transparency in reporting. For further information on Nature Research policies, see Authors & Referees and the Editorial Policy Checklist.

Statistics

For all statistical analyses, confirm that the following items are present in the figure legend, table legend, main text, or Methods section.

n/a	Confirmed	
☐	☒	The exact sample size (n) for each experimental group/condition, given as a discrete number and unit of measurement
☐	☒	A statement on whether measurements were taken from distinct samples or whether the same sample was measured repeatedly
☐	☒	The statistical test(s) used AND whether they are one- or two-sided<br>*Only common tests should be described solely by name; describe more complex techniques in the Methods section.*
☒	☐	A description of all covariates tested
☐	☒	A description of any assumptions or corrections, such as tests of normality and adjustment for multiple comparisons
☐	☒	A full description of the statistical parameters including central tendency (e.g. means) or other basic estimates (e.g. regression coefficient) AND variation (e.g. standard deviation) or associated estimates of uncertainty (e.g. confidence intervals)
☐	☒	For null hypothesis testing, the test statistic (e.g. F, t, r) with confidence intervals, effect sizes, degrees of freedom and P value noted<br>*Give P values as exact values whenever suitable.*
☒	☐	For Bayesian analysis, information on the choice of priors and Markov chain Monte Carlo settings
☒	☐	For hierarchical and complex designs, identification of the appropriate level for tests and full reporting of outcomes
☒	☐	Estimates of effect sizes (e.g. Cohen's d, Pearson's r), indicating how they were calculated

Our web collection on statistics for biologists contains articles on many of the points above.

Software and code

Policy information about availability of computer code

Data collection

Illumina HiSeq 2500, Illumina HiSeq4000, Oxford Nanopore MinION MkIb using R9.4.1 (rev D) flow cells. All data acquisition software was standard and included with the sequencing machines.

Data analysis

Images were processed with FIJI (v1.52p)
Images were further processed with Adobe Photoshop and assembled in Adobe Illustrator (2020 version)
Basecalling of nanopore sequencing data was performed using guppy v3.2.2
Alignment of Nanopore sequencing data was performed using minimap2 v2.15
Alignment of Illumina sequencing data was performed using GSNAP (version 2019-09-12)
Aligned datasets (Nanopore & Illumina) were processed using SAMtools v1.9 and BEDtools v2.27.1
Splice Junctions were extracted using RegTools v0.2.0
Further processing of Nanopore data was performed using BAMreadcount v0.8
G-test statistics were calculated using the Perl module Statistics::Distributions::GTest v0.1.5
Visualization of data was performed using the R packages Gviz and GenomicFeatures v3.10
Additional sequencing visualization was performed with ggplot2 v3.3.0
Further processing of the Illumina data involved calling peaks using MACS2 (v2.1.2) and visualization using deepTools2 (v3.3.1)
Detection of m6A by dRNA sequencing is now codified (DRUMMER; https://github.com/DepledgeLab/DRUMMER)

For manuscripts utilizing custom algorithms or software that are central to the research but not yet described in published literature, software must be made available to editors/reviewers. We strongly encourage code deposition in a community repository (e.g. GitHub). See the Nature Research guidelines for submitting code & software for further information.

Data

Policy information about availability of data

All manuscripts must include a data availability statement. This statement should provide the following information, where applicable:

- Accession codes, unique identifiers, or web links for publicly available datasets
- A list of figures that have associated raw data
- A description of any restrictions on data availability

Fast5 (Nanopore) and fastq (Illumina) datasets generated as part of this study can be downloaded from the European Nucleotide Archive (ENA) under the following study accession: PRJEB35652. The authors declare that all other data supporting the findings of this study are available within the article and its Supplementary Information files, or are available from the authors upon request. The source data underlying Figs 1a, 2d-f, 5, 6a-b, 6d-I, and 7b-f and Supplementary Figs 3b-h, 4a-b, 5a-e, 6a-j and 7b are provided as a Source Data file.

Field-specific reporting

Please select the one below that is the best fit for your research. If you are not sure, read the appropriate sections before making your selection.

☒ Life sciences ☐ Behavioural & social sciences ☐ Ecological, evolutionary & environmental sciences

For a reference copy of the document with all sections, see nature.com/documents/nr-reporting-summary-flat.pdf

Life sciences study design

All studies must disclose on these points even when the disclosure is negative.

Sample size	Sample size was not statistically determined. Methylated RNA Immunoprecipitation and sequencing experiments were composed of n=3 biological replicates for each mock-infected or adenovirus-infected sample. Standard Illumina sequencing was performed with n=3 biological replicates for adenovirus-infected control siRNA treated cells or siMETTL3 treated cells. Nanopore direct RNA sequencing was performed with n=2 biological replicates for METTL3 knockout A549 cells and n=2 biological replicates for wildtype A549 cells. These sample sizes were chosen because they are typical for similar sequencing experiments. Depth of sequencing was high for all runs, and the variation between runs was minimal as tested by principal component analysis (data not shown). All cell biology experiments were repeated at least 3 times, and are detailed in the methods and the figure legends.
Data exclusions	No data was excluded from this study.
Replication	Rigor and reproducibility were considered when designing the experiments in this study. Standard protocols and reagents were used for sample preparation and all sequencing experiments. All biochemical experiments were repeated at least three times with reproducible results.
Randomization	No randomization was performed for this study as most analysis was performed computationally and is not open to interpretation or user bias. Other assays such as Immunoblot analysis cannot be randomized.
Blinding	The experiments were not blinded as they were performed in an unbiased manner by algorithms and computer programs.

Reporting for specific materials, systems and methods

We require information from authors about some types of materials, experimental systems and methods used in many studies. Here, indicate whether each material, system or method listed is relevant to your study. If you are not sure if a list item applies to your research, read the appropriate section before selecting a response.

Materials & experimental systems

n/a	Involved in the study	
☐	☒	Antibodies
☐	☒	Eukaryotic cell lines
☒	☐	Palaeontology
☒	☐	Animals and other organisms
☒	☐	Human research participants
☒	☐	Clinical data

Methods

n/a	Involved in the study	
☒	☐	ChIP-seq
☒	☐	Flow cytometry
☒	☐	MRI-based neuroimaging

Antibodies

Antibodies used	METTL3 (Novus 577 Biologicals H00056339, WB: 1:400, IF: 1:50)<br>METTL14 (Sigma-Aldrich HPA038002, WB: 1:5000, IF: 1:100)<br>WTAP (Proteintech 60188, WB: 1:400, IF: 1:100)

	YTHDC1 (Abcam ab122340, WB: 1:2000, IF: 1:100)<br>YTHDF1 (Abcam ab99080, WB: 1:500, IF: 1:100)<br>YTHDF2 (Proteintech 24744-1-AP, WB: 1;1000, IF: 1:100)<br>YTHDF3 (Sigma-Aldrich SAB2102735, WB: 1:500)<br>FTO (Abcam ab92821, WB: 1:300, IF: 1:100)<br>ALKBH5 (Sigma-Aldrich SAB1407587, WB: 1:250, IF: 1:100)<br>Actin (Sigma-Aldrich A5441-100UL, WB 1:5000)<br>GAPDH (GeneTex 583 41577, WB:1:20,000)<br>RNA Pol II p-Ser2 (Abcam ab5095, IF: 1:400)<br>Flag (Sigma-Aldrich F7425-.2MG, WB 1:2000)<br>rabbit polyclonal against adenovirus Hexon, Penton, and Fiber (Non-commercial Gift from J. Wilson, PMID: 8673104, WB 1:10,000)<br>mouse anti-DBP (Non-commercial Gift from A. Levine, Clone: B6-8, PMID: 6310869, WB 1:1000, IF 1:400)<br>polyclonal rabbit anti-DBP (Non-commercial Gift from A. Levine, PMID: 6310869, IF: 1:40,000)<br>mouse anti-E1B55K (Non commercial Gift from A. Levine, Clone: 58K2A6, PMID: 7048730, WB 1:500)<br>mouse anti-E1A (BD 554155, WB: 1:500)<br>anti-mouse Alexa-fluor 488 (Invitrogen A-11001 IF 1:500)<br>anti-rabbit Alexa-fluor 488 (Invitrogen A-11008 IF 1:500)<br>anti-mouse Alexa-fluor 555 (Invitrogen A-21422 IF 1:500)<br>anti-rabbit Alexa-fluor 555 (Invitrogen A-21428 IF 1:500)<br>anti-mouse Alexa-fluor 568 (Invitrogen A-11004 IF 1:500)<br>anti-rabbit Alexa-fluor 568 (Invitrogen A-11011 IF 1:500)
Validation	Commercially available antibodies were validated by the manufacturer via Immunoblot or Immunoflourenscence Imaging. Non-commercial antibodies provided as a gift by other laboratories were validated in the original publications referenced in our methods section. In addition, we validated the specificity of these antibodies against cellular targets by immunoblot in the presence of siRNA targeting the specific gene, or for antibodies against viral targets by comparing mock-infected to infected cell lysate.

Eukaryotic cell lines

Policy information about cell lines

Cell line source(s)	A549 cells (ATCC CCL-185), HeLa cells (ATCC CCL-2) and HEK-293 cells (ATCC CRL-1573) were purchased from ATCC.
Authentication	All cell lines were purchased from ATCC without independent authentication.
Mycoplasma contamination	All cell lines tested negative for mycoplasma.
Commonly misidentified lines<br>(See ICLAC register)	No commonly misidentified cell lines were used.
