# Supplementary material for: Direct RNA sequencing reveals m6A modifications on adenovirus RNA are necessary for efficient splicing
Source: Nat Commun. 2020 Nov 26;11:6016. doi: 10.1038/s41467-020-19787-6 (PMC7691994; doi:10.1038/s41467-020-19787-6)
Supplement: Supplementary file 4 — Description of Additional Supplementary Files [file 41467_2020_19787_MOESM4_ESM.pdf]

Description of Additional Supplementary Files

File Name: Supplementary Data 1
Description: Table containing Illumina sequencing statistics for meRIP and RNA-seq experiments.

File Name: Supplementary Data 2
Description: meRIP-seq derived MACS2 peak locations for mock-infected cellular genes, Ad5-infected cellular genes, viral genes, and peak widths.

File Name: Supplementary Data 3
Description: Table containing Nanopore direct RNA sequencing statistics for experiments.

File Name: Supplementary Data 4
Description: Table containing exact locations of exome-level direct RNA sequencing analysis of m6A locations within viral transcripts.

File Name: Supplementary Data 5
Description: Table containing exact locations of isoform-level direct RNA sequencing analysis of m6A locations within viral transcript isoforms.
